# ECG Phenomena of the Early Ventricular Repolarization in the 21 Century

**Published:** 2008-08-01

**Authors:** Ihor Gussak, Samuel George, Bosko Bojovic, Branislav Vajdic

**Affiliations:** NewCardio, Inc, Santa Clara, CA, USA; University of Medicine and Dentistry-Robert Wood, Johnson Medical School, New Jersey, USA

**Keywords:** Early Ventricular Repolarization, Syndromes

## Introduction

Clinical interest in electrocardiographic (ECG) phenomena of early ventricular repolarization (EVR) has been rekindled recently mainly because of their clinically established association with fatal cardiac arrhythmias, particularly in otherwise healthy individuals with no (or minimal) structural diseases of the heart. ECG phenomena of EVR have often been misdiagnosed, misinterpreted, or undermined.  This happened mainly because of prevailing opinion of the"benign" or "misleading" nature of various EVR phenomena. For instance, early repolarization changes consistent with Brugada syndrome have been interpreted "innocent" and overlooked for decades until 1992 [1]. Another example - so-called "early repolarization syndrome" (ERS) that universally and unequivocally has been regarded as "normal", "normal variant", "benign early repolarization" until 2000 [2].  In 2008, seminal article by Haïssaguerre et al [3]. accompanied by editorial comments by Wellens [4] and letter to the editor by Nam et al [5]. brought clinical attention to an increased prevalence of the ERS among patients with a history of idiopathic ventricular fibrillation (VF).

The ECG phenomena of EVR include (but not are limited to):

- J-deflections
- "Slurring or notching" of the QRS complex (with or without J-deflections)
- J-waves (with or without ST-segment elevation).

The clinical validity of ECG manifestations of EVR as makers of sudden cardiac death (SCD) and their utility in risk stratification are not well defined. In addition, it is common for such ECG phenomena to vary significantly depending upon a variety of intrinsic cardiac and extracardiac factors. The main focus of this review is to characterize the ECG *patterns of the EVR* with a special focus on the ERS and its arrhythmogenic potential.

Ventricular repolarization begins when ventricular depolarization ends. In the normal heart, the evolution of depolarization into repolarization is relatively short process, and the magnitude of the overlap between the latest depolarization and the earliest repolarization does not exceed 10 msec [6]. The duration of this temporal overlap between the end of depolarization and the beginning of repolarization is greatly influenced by various physiological and pathological, cardiac and extracardiac conditions that affect either: (a) propagation of the excitation wave through ventricular wall or (b) recovery of its excitability, or (c) both. Among intrinsic cardiac factors are:

- An intrinsic configuration of the early phase of the actions potentials in different ventricular layers (endomyocardium, midmyocardium, and epicardium)
- A modification of the time-course of repolarization across the ventricular wall (transventricular vector gradient).

In a normal ECG, the transition of ventricular depolarization into ventricular repolarization corresponds on the surface ECG to the ***J-point***. The J-point defined as the point at which there is abrupt transition from the QRS complex to the ST-segment. Deviation of the J-point from the isoelectric line leads to the presence of a ***J-deflection***, which is a common ECG feature of ERS, but also seen in acute myocardial ischemia, hypercalemia, and various intraventricular conduction disturbances. A J-deflection inscribed on either the downsloping limb of the QRS complex or S wave is commonly regarded as a ***QRS-notching***, whereas a smooth and prolonged transition from the QRS segment to the ST segment - ***QRS-slurring***. Increased amplitude and duration of the J-deflection that takes the shape of a 'dome or a hump" is usually described as a ***J-wave*** [6].

Noteworthy to emphasize that a clear distinction between the delayed conduction and the EVR cannot always be made on the basis of an ECG alone. Nevertheless, distinguishing the two is very important since commonly EVR presents greater arrhythmia risk than a delayed intraventricular conduction, especially in otherwise healthy individuals. To limited extent, depolarization and repolarization processes can be differentiated based by their differences with respect to the heart rate, different cardiac drugs, and neurotransmitters (see below). The signal-averaged ECG could be helpful diagnostic tool but of limited clinical value in many cases [71], and the search for new ECG tools and markers to differentiate these two processes is accelerating.

## "Slurring or Notching" of the QRS Complex

In 1998, Garg and his associates [8] reported a case of "familial sudden cardiac death associated with a terminal QRS-abnormality on surface 12-lead electrocardiogram". The abnormal low-amplitude deflections in the downsloping limp of the QRS complex in leads II, III, aVF and I, aVL and V6 were coincident with the late potential on the signal-averaged ECG. This deflection appeared to be more prominent after procainamide or beta-blockers and normalized after quinidine. Moreover, sustained polymorphic ventricular tachycardia, which degenerated to VF was easily inducible during programmed stimulation from the right ventricular apex, despite administration of procainamide or atenolol. The clinical significance of these ECG findings is not fully understood at present and further investigation is warranted.

## J-Waves

Different names have been used at different time for the J-wave. They include "camel hump sign","hathook junction", "K wave", "H wave", "late delta wave", "current of injury", "J point wave", "hypothermic wave", "hypothermic hump", "Osborn wave".  J-waves can be classified as:

- Hypothermic
- Non-hypothermic
- Idiopathic.

### Hypothermic J-waves

Clinical as well as experimental data linking hypothermic J-waves and cardiac arrhythmias remain sparse and somewhat contradictory; the occurrence of ventricular tachyarrhythmias associated with hypothermic J-wave varies from 0 to almost 100% [9]. Sodium channel blockers, such as procainamide and lidocaine, are ineffective, indeed proarrhythmic in both the prevention and treatment of the malignant ventricular tachyarrhythmias in hypothermic patients during their rewarming [9].

### Non-hypothermic J-Waves

ECG changes resembling those in hypothermia-induced J-waves have been observed in a various clinical and experimental settings with normal body temperature, such as acute myocardial ischemia, acute pulmonary thromboembolism, right ventricular infarction, electrolyte or metabolic disorders, pulmonary or inflammatory diseases, or abnormalities of central or peripheral nervous system, intoxication by heterocyclic antidepressant or cocaine, and many other abnormal conditions [10]. Among those clinical situations, prominent J-waves most frequently are observed in acute myocardial ischemia and hypercalemia, and their arrhythmogenic potentials in most clinical settings are chiefly dependent upon the underlying disease.

### Idiopathic J-Waves

In the absence of any structural cardiac abnormalities or extracardiac diseases, changes of EVR can be classified as primary or "idiopathic". Several forms of idiopathic appearance of a J-wave in human with or without accompanying ST-segment elevation have been described. Among them, the most investigated is so-called ECG marker of Brugada syndrome. Idiopathic J-wave followed by downsloping ST-segment elevation with inverted T-waves in the right chest leads is an ECG hallmark of the "typical" Brugada syndrome.

However, in many clinical cases of symptomatic Brugada syndrome (atrial and/or ventricular tachyarrhythmias or SCD), the pattern of ST-T abnormalities and/or their ECG "localization" is different from that in the "typical" Brugada Syndrome. In such cases, the terms "atypical" or "J-wave - like' are appropriate. Clinically noteworthy that J-wave-like ECG abnormalities in leads other that the right precordial (e.g. inferior or lateral) ("atypical" Brugada syndrome) have been also described in otherwise healthy individuals prone to paroxysmal ventricular tachycardia/fibrillation.

## Early Repolarization Syndrome

The term "early repolarization syndrome" was introduced nearly half a century ago and has traditionally been regarded as idiopathic and benign or "innocent" [11] or "misleading" [12] ECG pattern of EVR until 2000 [2]. The ERS has been ascribed a number of names, including "early repolarization", "early ventricular repolarization', 'benign early repolarization", "benign J wave", "nonspecific changes of ventricular repolarization", "repolarization variant", "normal variant RS-T segment elevation", and "juvenile or unconventional ST-T pattern" and cetera to describe the "characteristic" ECG pattern of J-deflection followed by horizontal ST-segment elevation in the mid-precordial leads [2].

To avoid further confusion and inconsistencies with terminology, we prefer to use the term "early repolarization syndrome", acknowledging at the same that this term is not the best one to use in the cases of asymptomatic forms of EVR. By its own definition, any clinical syndrome is a combination of signs and symptoms that occur together and characterize a particular abnormality. The term "syndrome" is best reserved for a description of clinical manifestation of the disease. In this context, the "true ERS" is best defined as an arrhythmogenic entity that is characterized by (a) ECG marker of ERS that is associated with (b) arrhythmogenic complications, including SCD, and/or family history of SCD in otherwise healthy individual.

The prevalence of ERS varies between 1% [13] and 2% [14].  It is more commonly seen in young individuals (27.5%) [15], especially those predisposed to vagotonia, and shows a clear *male preponderance* (77%) [13]. The syndrome is also often observed in:

- Athletes [16]
- Cocaine users [17]
- Obstructive hypertrophic cardiomyopathy [18]
- Defects and/or hypertrophy of the interventricular septum [18].

The electrocardiographic manifestation of ERS is often dependent on heart rate, normalizing during exercise or with rapid pacing, as well as with advancing age [19]. Familial occurrence of the syndrome has been observed [20,21]. Although early studies were interpreted to suggest that the syndrome is more prevalent in the Black population, more-recent studies challenge this notion [22].

In 2000, based on preclinical experimental work from Dr. Charles Antzelevitch, it has been suggested that: (a) ERS should not be considered as normal or benign ECG abnormality *a priori*, unless otherwise proven, as generally thought and (b)  under certain conditions known to predispose to ST-segment elevation, patients with ERS may be at greater arrhythmogenic risk [2].

The classical ECG pattern of ERS consists of: (a) prominent notch or slur on the downsloping portion of the QRS complex, or J-deflection, (b) followed by diffuse upward ST segment concavity concordant with the QRS complex, and (c) positive T wave in the same lead. Additional ECG feature include:

- *Localization* of the ECG pattern of ERS in scalar ECG. Mid-to-lateral precordial leads V_2_-V_4(5)_ have been recognized as showing the most prominent repolarization changes consistent with ERS. Noteworthy, similar changes might appear in other leads but to a lesser extent
- Reciprocal ST segment depression in aVR
- "Waxing and waning" of the ST-T segment over time
- Often, ERS is associated with shorter-than-normal QT interval duration, even adjusted to the heart rate.

Nevertheless, in many clinical instances, it is still very difficult to distinguish subjects with ERS from those with the Brugada syndrome or various intraventricular conduction blocks, or Short QT syndrome based solely on a resting ECG. Furthermore, the ECG alterations in response to changes in heart rate, drugs effects, and autonomic tone observed in ERS are very similar to those observed (a) under hypothermic conditions and (b) the Brugada syndrome (Table 1).

Many clinical features of ERS are similar to that of Brugada syndrome, including (a) predominance in young otherwise healthy males, (b) predisposition to familial occurrence, (c) in many individuals, a transient normalization of ECG manifestation, and (d) ERS similar responses to drugs and autonomic modulation.

Two major features permit differentiation of the ECG signatures of ERS and the Brugada syndrome: (a) pattern and (b) leads specificity. The elevated ST-segment in ERS is usually localized in leads  V_2_-V_4(5)_ and has an upward concavity with positive T-wave polarity accompanied by a notched (or slurred) J-deflection.  In contrast, the ECG of Brugada patients generally displays a prominent J-point elevation (J-wave), followed by a downsloping ST segments and negative T-wave in the right precordial leads V_1_-V_3_ ("typical") or other leads ("atypical").

It is often difficult to distinguish subjects with ERS from those with the Brugada syndrome, especially when the latter also display intraventricular conduction slowing or block. In 1993, Aizawa and his colleagues [23] described several patients with idiopathic ventricular fibrillation in whom they found "bradycardia-dependent *intraventricular block*". The common ECG features of these patients included: (a) incomplete right bundle branch block, (b) prominent "notch" on downsloping limb of the QRS complex in leads V_3_-V_5_, II, III, and aVF, (c) elevated ST segment with positive T waves leads V_2_-V_3_, and (d) rate (deceleration) - dependent accentuation of the prominence of the "notch".

The dynamicity of ECG changes in ERS and Brugada syndrome is also confounding. When the clinical history is malignant, an invasive electrophysiological study examining the inducibility of ventricular tachycardia/fibrillation may be useful in assessing arrhythmogenic risk.

Interestingly, ECG changes consistent with the Brugada syndrome have also been referred to as a benign repolarization variant or "Ideiken phenomenon" for more than three decades and some patients having such ECG abnormalities have been described as "unwitting victims of electrocardiography" [2].

No doubt, the intrinsic capability of the standard 12-lead ECG are significantly limited in the differential diagnosis of the ERS, since only a small portion of the available diagnostic information can be obtained from conventional scalar 12-lead ECG tracings. The search for new additional and more advanced and sophisticated computer-assisted methodologies and ECG markers that can help to differentiate ST-segment elevations in ERS from other ECG phenomena of EVR (e.g. Brugada syndrome, acute ischemia) have intensified in most recent years. The "three-dimensional (3D) approach" is expected to be valuable in EVR evaluation. Some of those 3D - ECG markers (e.g. ST-T and QRS-T angles) are already in their "clinical validation" stages of development (1).

### Possible Cellular and Ionic Mechanisms

Concordant with the QRS complex, ST segment elevation is most commonly recognized as a sign of acute myocardial damage, often associated with the development of cardiac arrhythmias. The electrophysiological nature of the ST-segment elevation in ERS, whether idiopathic or due to so-called "current of injury", has been investigated by means of a direct-current magnetocardiogram, which, in contrast to conventional 12-lead ECG, is capable of determining a TQ interval shifts. The results showed clearly that ST-segment shifts in subjects with ERS are unrelated to ischemic injury [24].

One might also explain the principal difference between ERS and Brugada syndrome based on an abbreviation of the action potential due to loss of its "dome" resulting in the development of a very significant transmural as well as epicardial dispersion of repolarization and refractoriness, setting the stage for both phase 2 and circus movement reentry culminating in much more severe arrhythmogenic manifestations of the Brugada syndrome if compared with ERS [2].

Some believe that parietal (myocardial) conduction defects (delayed conduction) could contribute to both the ECG manifestation and the arrhythmogenic potentials of the different ECG phenomena of EVR.

### Modulation by Drugs, Rate, and Neurotransmitters

The changes in the magnitude of the EVR abnormalities in ERS and Brugada syndrome, like a hypothermic J wave, display qualitatively similar responses to a variety of drugs as well as to changes in rate and autonomic tone (Table 1) [2]:

- *Slowing of heart rate* exaggerates J-waves and ST-segment elevation, while increase in heart rate during exercise or following isoproterenol reduces or even eliminates these ECG abnormalities
- *Sodium channel blockers* (known to unmask the Brugada syndrome) increase ST-segment elevation in ERS subjects and hypothermic J-waves
- *Sympathetic stimulation and β-adrenergic* agonists normalize the ST-segment in both syndromes, whereas β-adrenergic blockers augment ST-segment elevation in both, while propranolol increases its magnitude and its toxicity may even induce classical pattern of ERS.

### The Role of Nervous System

Clinical and experimental studies point to high spinal cord injury as a cause of ERS-like changes in the ECG.  High cervical spinal chord injury can lead to significant deterioration or even complete disruption of the cardiac sympathetic activity, leaving parasympathetic activity unopposed [25]. Parasympathetic activation has an opposite effect in both syndromes, causing ST segment elevation due to depression or loss of the action potential plateau (see below). ECG patterns "wax and wane" in both syndromes, possibly due to variations of autonomic activity [2].

### Arrhythmogenic Potential

In vast majority of cases, ERS *is not a malignant* EVR syndrome per *se, unless otherwise proven*. However, the similarities between the Brugada syndrome and ERS in response to rate, neuromodulation and pharmacologic agents strongly suggest a parallelism of mechanisms.

While ERS has long been considered to be benign, in experimental models, the ECG signature of ERS can be ***converted*** to that of the Brugada syndrome [2,26,27]. This raises the possibility that ERS *may not be as benign (unless otherwise proven)* as generally believed, and that under certain conditions known to predispose to ST-segment elevation, ERS subjects may be at increased risk.  In considering these possibilities, it is instructive to remember that (a) the Brugada syndrome was regarded as benign for more than three decades, and (b) that one syndrome can be readily converted to the other in experimental models involving the wedge preparation.

One hypothesis presumes that depression of the epicardial, but not endocardial, action potential plateau creates a transmural gradient that manifests on the scalar ECG as a complex ST-segment elevation with a positive T-wave. As such the cellular substrate is not arrhythmogenic, but in case of further increase in net repolarizing current as a result of complete loss of the epicardial action potential dome and the attendant development of a large transmural dispersion of repolarization could become arrhythmogenic. This proposed mechanism also explain the highly arrhythmogenic character of the Brugada syndrome and apparently benign exceptions.

Alternative hypothesis is based on assumption that the delayed (parietal) intraventricular conduction delay in the proximity of interventricular septum (mid-precordial leads could contribute to the ECG phenomenon of ERS and its potential link to electrical instability.

It is important to emphasize that these hypotheses remain to be more rigorously tested and that the distinct nosologic entity that is referred as the ERS needs to be more fully delineated within the framework of what we have learned about the Brugada syndrome in recent years. A careful clinical history and invasive electrophysiological studies may be required to determine whether or not the early ventricular repolarization abnormalities in a given patient are benign or malignant.

## Summary, Clinical Implementation, and Future Directions

The available clinical data suggest that the different ECG phenomena of the EVR syndromes often share similar ECG presentations, mechanisms with wide range of their arrhythmogenic potentials. ECG similarities between different ECG phenomena of EVR raise certain concerns for their misdiagnosis and clinical relevance.

In our opinion:

1. ERS, as a diagnosis, should not be regarded as either benign or malignant *a priori* unless otherwise proven.
2. Clinical judgment based on clinical presentation ("arrhythmogenic" anamnesis), family history or syncope/SCD, potential use of cardio-active drugs, including psychotropic medications is the most essential in the risk stratification of the ERS subject. Special attention should be devoted to the family history of primary EVR abnormalities and/or SCD.
3. Additional diagnostic work-up, such as tilt-test, signal-averaging ECG, and electrophysiological studies with or without drug testing, should be considered on the case-by-case basis upon physician's discretion. Genetic screening of ERS subjects at risk, once available, will be one of the most important diagnostic tools to identify and/or confirm the diagnosis of primary electrical abnormalities/diseases of the EVR.
4. It is important to keep in mind that ERS subjects might be predisposed to the drug-induced ventricular arrhythmias.
5. Standard 12-lead ECG is very valuable diagnostic tool to identify ERS subjects, yet its clinical utility in the risk stratification is of limited intrinsic value. New ECG technologies, methodologies, and markers should be developed and clinically validated to help to identify and differentiate ERS from other forms of EVR as well as to stratify this risk.

## Figures and Tables

**Figure 1:**
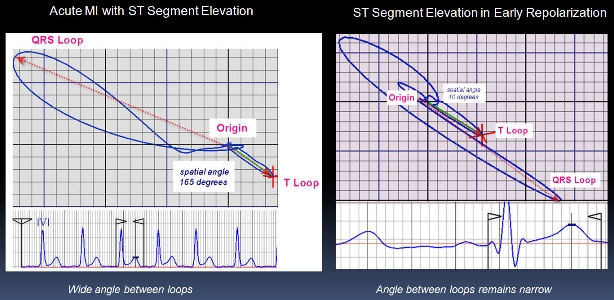
Acute ischemia induces wide angle between QRS and T vector loops, but the angle remains narrow in ST elevation associated with early repolarization.  In acute myocardial ischemia, the QRS and T loops become widely divergent and the spatial angle between them is greater than 75º in a significant majority of cases.  In contrast, the angle is unaffected in early repolarization (from http://www.newcardio.com/)

**Table 1:**
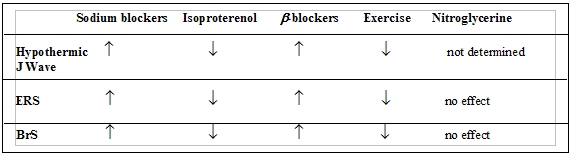
Autonomic and pharmacologic modulation of the J-wave magnitude under hypothermic conditions, in the early repolarization (ERS) and Brugada syndromes (BrS).
